# Occurrence and Fate of Fluoroalkyl Sulfonamide-Based Copolymers in Earthworms–Bioavailability, Transformation, and Potential Impact of Sludge Application

**DOI:** 10.1021/acs.est.4c01844

**Published:** 2024-10-04

**Authors:** Felicia Fredriksson, Anna Kärrman, Ulrika Eriksson, Leo WY Yeung

**Affiliations:** Man-Technology-Environment (MTM) Research Centre, School of Science and Technology, Örebro University, Orebro SE-701 82, Sweden

**Keywords:** per- and polyfluoroalkyl substances (PFAS), FASA-based copolymers, biotransformation, bioaccumulation, earthworms, sludge-amended soil

## Abstract

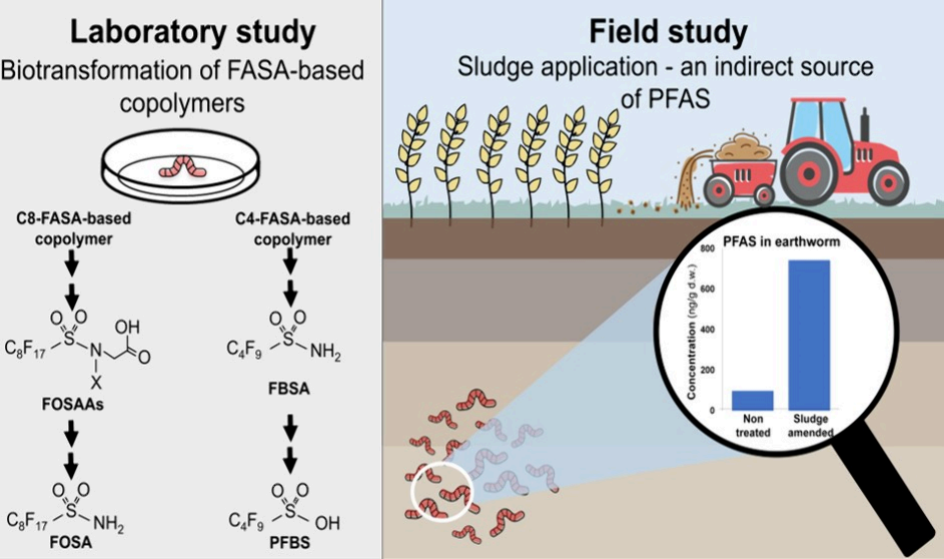

To date, considerable knowledge and data gaps regarding the occurrence, environmental levels, and fate of polymeric perfluoroalkyl and polyfluoroalkyl substances (PFAS) exist. In the present study availability, accumulation, and transformation of C4- and C8-fluoroalkylsulfonamide (FASA)-based copolymers were assessed in laboratory-grown earthworms (*Eisenia fetida*, triplicate of exposure tests and control). Further, a field study on earthworms (18 pooled samples) in sludge-amended soil was conducted to assess the environmental impact of sludge-amended soil with regard to the FASA-based copolymers, together with the applied sludge (*n* = 3), and the field soils during the period (*n* = 4). In the laboratory study, the FASA-based copolymers were taken up by the earthworms in concentrations between 19 and 33 ng/g of dw for the C8- and between 767 and 1735 ng/g of dw for the C4-FASA-based copolymer. Higher biota soil accumulation factors (BAFs) were observed for the copolymer with a longer perfluorinated side-chain length (C8, average BAF value of 0.7) compared to the copolymer with a shorter side-chain length (C4, average BAF value of 0.02). Perfluorooctane sulfonamidoacetates (FOSAAs) and perfluorooctane sulfonamide (FOSA), including both branched and linear isomers, were detected after exposure to the C8-FASA-based copolymer. Two metabolites were detected in the earthworms exposed to the C4-FASA-based copolymer: perfluorobutanesulfonamide (FBSA) and perfluorobutanesulfonic acid (PFBS). Although the presence of other monomers or impurities in the copolymer formulation cannot be ruled out, the present laboratory study suggests that the FASA-based copolymers may be an indirect source of lower molecular weight PFAS in the environment through transformation. Elevated levels of C8-FASA-based copolymer were found in the field sludge-amended soil compared to nontreated soil (32 versus 11 ng/g d.w.), and higher concentrations of PFAS in earthworms living in sludge-amended soil compared to nontreated soil (566 versus 103 ng/g d.w.) were observed. These findings imply that the application of sludge is a potential pathway of PFAS to the environment.

## Abstract 2

This study suggests FASA-based copolymers to be an overlooked indirect source of low molecular PFAS in the environment.

## 1 Introduction

Per- and polyfluoroalkyl substances (PFAS) are an ongoing concern for the environment, wildlife, and human health. Environmental research on PFAS has mainly been focused on perfluoroalkyl acids (PFAAs), due to their persistence, toxicity, bioaccumulation potential, and global occurrence in the environment.^1−5^ Besides this compound class, the PFAS family consists of a wide range of anthropogenic compounds with a large chemical diversity,^6^ where numerous PFAS have been detected in various products and environmental matrices.^7−11^ There are considerable knowledge and data gaps regarding newly identified PFAS in terms of their occurrence, environmental levels, and fate, especially in regard to bioavailability, bioaccumulation, and biotransformation. Ubiquitous occurrences of PFAAs have been found in several biota matrices from both terrestrial and aquatic systems.^12−16^ The detection of PFAAs in biota may be a result of the extensive historical use or an indirect source through transformations of other precursor compounds and their bioaccumulation potentials. Several newly identified PFAS are potential precursors to PFAAs in the environment, such as PFAS with tertiary amine or quaternary ammonium groups,^17^ polyfluoro-betaines,^18^ and side-chain fluorinated polymers.^19^

At the moment, limited studies on the environmental fate of fluorinated polymers have been conducted, even though one of the largest shares of PFAS on the global market are fluorinated polymers.^20^ Side-chain fluorinated polymers haveper-/polyfluorinated side chains attached to a nonfluorinated polymeric backbone.^1^ One of the primary producers of PFAS, the 3M Company, phased out products based on perfluorooctanyl (C8) chemistry in early 2000^21^ and replaced it with shorter-chain homologues, including the active ingredients in textile impregnation products from the trademark Scotchgard. In the beginning of 2023, the 3M Company announced the phase-out of PFAS in Scotchgard products, and by 2025, they will discontinue manufacturing PFAS. However, since there is currently no internationally agreed definition of PFAS, how the company defines PFAS is not clarified. Whether fluorinated compounds will be completely phased out by 2025 is yet to be known.^22^ Detection of suspected side-chain fluorinated copolymers used in Scotchgard formulations before and after 2002 (pre-2002 and post-2002) were observed to differ in fluorinated side-chain lengths; C8 chain (i.e., ethyl-perfluorooctane sulfonamide, EtFOSA) were used before 2002 and was replaced with C4 chain (i.e., methyl-perfluorobutane sulfonamide, MeFBSA) after the phase-out of perfluorooctanesulfonyl fluoride (POSF).^23^ Our recent study proposed the molecular structure in the pre-2002 formulation to be a C8-fluoroalkyl sulfonamide (FASA)-based copolymer (CAS no: 21055–88–9).^24^ The exact molecular structure of the post-2002 formulation was not identified. However, the copolymer has been discovered to contain MeFBSA moiety(ies) and a mass-to-charge of 1634.30548 in negative electrospray ionization mode,^23^ the component is herein called C4-FASA-based copolymer.

The C4- and C8-FASA-based copolymers have been found in different matrices such as biosolid-augmented soil, sediments, biosolids, and sludge.^25−27^ Recent results from a sorption test indicate that these FASA-based copolymers are readily sorbed onto particles.^24^ Even though they are readily sorbed to soil, we would like to examine if these copolymers might be available to terrestrial organisms (e.g., earthworms) when exposed to sludge-amended agricultural soil. Uptake by earthworms in biosolid-amended soil for other types of PFAS has been noted.^28−30^ Thus, it is important to understand the potential environmental pathways and bioavailabilities of these FASA-based copolymers. This study aimed to deepen our knowledge on the occurrence and fate of the two FASA-based copolymers in soil-earthworm systems. Laboratory-grown earthworms (*Eisenia fetida*) were exposed to FASA-based copolymer-fortified soil to evaluate their bioavailability, bioaccumulation, and biotransformation. Further, the environmental fate of FASA-based copolymers in a sludge-amended agricultural field was studied to understand their potential to be an indirect source of PFAA contamination.

## 2 Materials and Methods

### 2.1 Chemicals

Technical mixtures of Scotchgard pre-2002 and post-2002 formulations (1000 μg/mL in methanol) were used for the exposure study and purchased from AccuStandard Inc. (New Haven, USA). Both technical mixtures were used in the exposure study without any purification. Native and mass-labeled PFAS standards used for the target analysis including perfluoroalkyl sulfonic acids (PFSAs), perfluoroalkyl carboxylic acids (PFCAs), FASAs, perfluorooctane sulfonamidoacetates (FOSAAs), and fluorotelomer sulfonic acids (FTSAs) were obtained from Wellington Laboratories (Guelph, Canada). Further details on target analytes, mass-labeled standards, chemicals, and materials are provided in the Supporting Information (SI).

### 2.2 Laboratory Study

#### 2.2.1 Soil and Test Organisms

An artificial soil was prepared according to standard protocols (OECD 317, ISO 11268–1)^31,32^ since the presence of micropollutants can influence the results, and noncontaminated natural soils were not available for the bioavailability test. Further details on soil preparation are provided in the SI. For the laboratory study, mature earthworms (*Eisenia fetida*) were cultivated in our laboratory following the guidelines of ISO 11268–1.^31^ No FASA-based copolymers or other target PFAS were detected in the earthworms.

#### 2.2.2 Experimental Design

**Figure 1:**
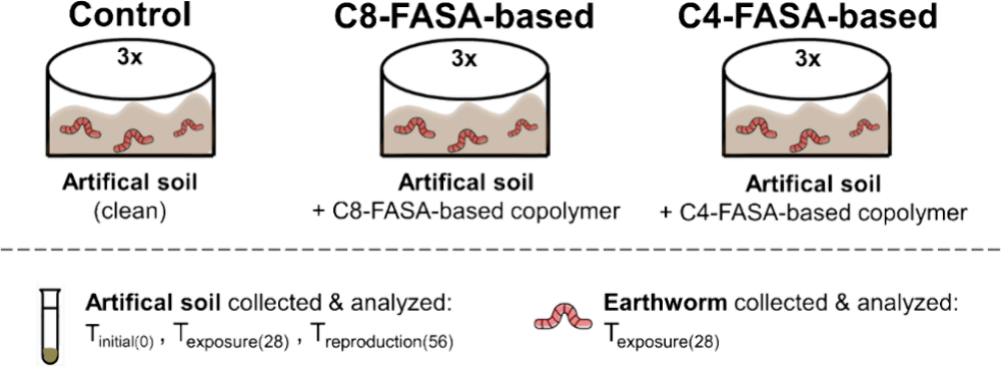
Experimental design showing the different treatment conditions across the three groups (control, C8-FASA-based, and C4-FASA-based), together with the steps of collection of samples (soil and earthworms).

To study the bioavailability and biotransformation of the FASA-based copolymers, a series of glass vessels containing 500 g of artificial soil fortified with the technical mixtures of pre-2002 or post-2002 formula (1000 μg) was used (Figure 1). The test was conducted in triplicate for 28 days and was adapted from standard protocols and in-house protocols.^31,32^ Triplicates of nonspiked soil with earthworms were set up as blank controls and performed under identical conditions as the spiked soil. In each glass container, ten mature earthworms with a total weight of 4.8–5.0 g (Table S9) were added to the soil in a closed system. The soil layer was approximately 4 cm, and the burrowing behavior was monitored to ensure that the experimental conditions were appropriate. During the 28 day exposure experiment, the mass of each glass vessel was monitored twice a week, and the soil was moistened when necessary to compensate for water losses. The earthworms were fed weekly ground oat flakes. The food was tested for PFAS before usage, and no target PFAS was above LOD. A controlled light/dark cycle (16 h/8 h) at a fixed temperature of 20 °C was carried out throughout the test.

After 28 days of exposure, the laboratory earthworm’s growth and survival were evaluated before they were removed from the soil. The experiment was continued according to ISO 11268–2 in order to determine the effects on reproduction.^33^ The earthworms were washed with ultrapure water (18.2 MΩ) and allowed to depurate on clean moist filter paper for >24 h. From each test container, the earthworms were pooled together, resulting in triplicate for the control and the two exposure setups, respectively. The pooled earthworm samples (*n* = 9) were frozen overnight (<−20 °C) and freeze-dried. The freeze-dried earthworms were homogenized by cutting them into small pieces and ground using a mortar and pestle. Subsamples of the exposure soil from respective glass containers were freeze-dried and homogenized by grinding. Both soil and earthworm samples were stored at −18 °C until analysis.

### 2.3 Field Study

#### 2.3.1 Field Study Area and Sample Collection

The study area was an agricultural field in southern Sweden, where municipality sludge has been applied every fourth year since 1981 (i.e., 1981, 1985, 1989, 1993, 1997, 2001, 2005, 2009, 2013, 2017, 2021).^34^ The field site is an area of a larger agricultural field, and the sampling area is divided into plots (6 × 20 m) organized in grids, receiving 0-, 4-, and 12-ton sludge per hectare.^35^ During this study, sludge was applied in September 2021. Winter wheat was sown during the period when sludge was applied, and the harvest of the wheat was one year after application (09–2022). Earthworm and soil samples were collected from the plots receiving 0- and 12-ton sludge per hectare, and during three time points, all plots received the same amount of nitrogen, phosphorus, and potassium (NPK) fertilizer. The plots receiving 0 ton sludge per hectare correspond to normal agriculture, acting as a reference, versus the plots receiving 12 ton sludge per hectare attempt to simulate sludge supply over a longer period. The time points were: T1–a couple of weeks before application of sludge (08–2021), representing no sludge has been applied on the specific plots receiving sludge since 2017, simulating the present state of PFAS level of the field site; T2–2 weeks after the application of sludge (10–2021), corresponding to the effect of application of sludge; and T3–1 week after the harvest of the winter wheat (09–2022), simulating a longer period effect of sludge.

The sampling of earthworms was conducted by digging into the soil using a precleaned metal spade. The earthworms were collected and pooled together from plots in the grids that received 0- or 12-ton sludge per hectare in precleaned polyethylene containers containing the soil from the corresponding plots. The earthworms were directly delivered to the laboratory (<12 h) and washed with ultrapure water (18.2 MΩ) before they were allowed to depurate on clean moist filter paper for >24 h. The earthworms were frozen overnight (<−20 °C) and freeze-dried. The freeze-dried earthworms were homogenized by cutting and grinding. A soil sample (0–25 cm depth) from each plot was collected, sieved (<2 mm), freeze-dried, and homogenized by grinding. The applied sludge was collected in triplicate before application and was freeze-dried and homogenized by grinding. All samples (*n* = 25; earthworm, soil, and sludge) were stored at −18 °C until analysis.

### 2.4 Sample Extraction

The earthworms collected after the exposure test and at the field site were extracted for the C4- and C8-FASA-based copolymers and for the anionic and neutral PFAS (potential transformation products). Soil from the exposure test was extracted for the FASA-based copolymers and anionic and neutral PFAS. The abiotic samples (sludge and soil) from the field site were extracted only for the FASA-based copolymers. The extraction method targeting the FASA-based copolymers followed the procedure developed by Chu and Letcher^26^ with minor modifications. The method for extracting the anionic and neutral PFAS followed Koch et al.,^12^ with an additional cleanup using graphitized carbon SPE columns (ENVI-Carb). The extraction and cleanup procedures for the C4- and C8-FASA-based copolymers, and for the anionic and neutral PFAS are described fully in the SI.

### 2.5 Instrumental Analysis

Analysis of the target analytes with exception of ultrashort chain PFAAs was performed on an Acquity UltraPerformance Liquid Chromatograph (UPLC) system coupled to a XEVO TQ-S tandem mass spectrometer (MS/MS; Waters Corporation, Milford, USA). The ultrashort chain (C1–C3) PFAAs were analyzed with supercritical fluid chromatograph (SFC) coupled to a TQ-Sμ MS/MS (Water Corporation, Milford, USA). The instruments were operated in negative ionization mode with multiple reaction monitoring (MRM). For the FASA-based copolymers since their respectively fluorinated side-chain is known (i.e., EtFOSA for C8-FASA-based and MeFBSA for C4-FASA-based copolymer), the identification of these copolymers was based on the main detectable chromatographic peak as the precursor ion and respective product ions in which fluorinated fragment ions were shown as MRM transitions. Details of the underlying principles are provided elsewhere.^23,24^ To further investigate transformation products from the laboratory study, all extracts were analyzed on a Xevo G2-XS QTof Quadrupole Time-of-Flight mass spectrometer (Waters Corporation, Milford, USA). The analysis was conducted in full scan (*m*/*z* 50–2000) with both negative and positive electrospray ionization. Further information about all target analytes and instrument analysis settings is described further in the SI.

### 2.6 Quality Control and Assurance and Data Analysis

The large suite of anionic and neutral PFAS was quantified by internal calibration (Table S1). For the exposure test, a matrix match calibration was performed for the FASA-based copolymers since no suitable internal standards were available. To take into account both the matrix effect and the extraction efficiency, samples were spiked prior to extraction (*n* = 3), and a correction factor was applied to the concentration quantified with external calibration.

Standards used for the quantification of C4- and C8-FASA-based copolymers were quantified in samples using nominal gravimetric concentrations of the technical mixtures as reference standards are not commercially available. Quantification using the concentration of the technical mixture may give an overestimation because the percentage of the copolymers in the technical mixture was not known. Since the total fluorine content of the technical mixtures has been determined^24^ and both copolymers’ perfluoroalkyl chains have been identified,^23^ fluorinated side-chain equivalent (FSC eq) can be calculated for both copolymers to compare the levels of the copolymers with the other PFAS. The equivalent FSC eq was determined to be 0.8% of the technical mixture for the C8-FASA-based copolymer and 3.1% of the technical mixture for the C4-FASA-based copolymer.

The extraction methods for both earthworms and soil were evaluated by spiking experiments. During each extraction batch, at least one solvent and matrix blank were conducted. For validation of the anionic and neutral PFAS extraction procedure, one spiked matrix quality control (QC) sample was additionally performed for each extraction batch. For the earthworms, the matrix used for the blank and QC spike was obtained from earthworms cultivated in our laboratory that had not been exposed to any PFAS. For the soil, the matrix for blank and QC samples was the control soil used in the exposure test. The method validation is described in more detail in the SI.

The method detection limit (MDL) was determined as the average plus three times the standard deviation of the control tests for the laboratory study and procedure blanks for the field study. In the absence of an analyte in the procedure blanks, the lowest point of a seven-point calibration curve was used as the MDL. For the reported levels of anionic and neutral PFAS, a recovery range between 75 and 125% of the internal standard was set as acceptable for most of the analytes with some exceptions. A lower recovery of the internal standard was still accepted after confirmation with the QC spike with native standards which was within an acceptable range. All recoveries of quality control spike samples and internal standards can be found in the SI.

The bioaccumulation factor (BAF) was calculated for the earthworms after exposure to the FASA-based copolymer in each soil. This was done by dividing the level of the FASA-based copolymer in the earthworms by the level of the FASA-based copolymer in the soil; both concentrations were reported on a dry weight basis (see equation).
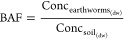


In the present study, no uptake kinetics of the FASA-based copolymers were measured, and no demonstration of steady-state conditions could therefore be made. The steady state was assumed to be reached. No accumulation factor was growth corrected. To assess the differences between the control groups and exposed groups, *t* tests were conducted.

## 3 Results and Discussions

### 3.1 Laboratory Study

After the exposure period, low mortality of the earthworms was observed in the test groups (0 to 3.3%, Table S9), which was within the validity of the test (<10%, OECD317, ISO 11268–1). No mortality was observed in the control test groups. The earthworms were fed weekly during the experiment and gained weight (Table S9). A reduced growth was observed for the earthworms exposed to C4- or C8-FASA-based copolymers (−24%; *p*-value <0.05; −26%, *p*-value <0.01, respectively) compared to the control. This observation suggests that exposure to the copolymers and/or their transformation products might affect the growth of the earthworms. Contamination of perfluorooctanesulfonic acid (PFOS) and perfluorooctanoic acid (PFOA) in soil has been reported to cause weight loss at exposure concentrations from 10 μg/g for PFOS^36^ and from 25 μg/g for PFOA.^37^ The exposure of the FASA-based copolymer may affect the reproduction since a lower rate of production of juveniles was seen in some of the groups exposed to C4- or C8-FASA-based copolymer compared to the control groups (Table S9; average −29% and −27% lower than the control). However, this could not be confirmed due to the low number of replicates and apparent variability. Further studies are necessary to confirm whether the copolymers may have any reproduction effect.

#### 3.1.1 Bioavailability of the FASA-Based Copolymer in Earthworms

The C4- or C8-FASA-based copolymer was found in the exposed earthworms after exposure for 28 days (Table S10). No FASA-based copolymers were detected in any of the earthworms nor soils of the control groups. Since the FASA-based copolymers were only a fraction of the technical mixture, the accumulated levels of the FASA-based copolymers were also expressed in FSC eq. The concentration of the C8-FASA-based copolymer in the earthworms ranged from 767 to 1735 ng/g dw (or 6.3 to 14 ng FSC eq/g dw), whereas the concentrations of the C4-FASA-based copolymer were in a range of 19 to 33 ng/g dw (or 0.58 to 1.0 ng FSC eq/g dw). All concentrations in the earthworms were measured after a depuration period of more than 24 h, assuming that the gastrointestinal tract of the earthworms was empty. No observed soil particles were noted on the outer skin of earthworms. The levels of the C8-FASA-based copolymer in the earthworms were up to 50 times higher than the C4-FASA-based copolymer. The higher abundance of the C8-FASA-based copolymer suggests a higher availability of the C8-FASA-based copolymer compared to the C4-FASA-based copolymer. Factors influencing the higher abundance might be a higher accumulation potential for the C8-FASA-based copolymer or the C4-FASA-based copolymer that might have a higher degradation rate/depurate rate/clearance rate compared to the C8-FASA-based copolymer resulting in a lower accumulation rate. Further studies are required to determine which factors influence the higher abundance of the C8-FASA-based copolymer compared to the C4-FASA-based copolymer in earthworms.

#### 3.1.2 Biotransformation of the FASA-Based Copolymers

The earthworms and soil from the exposure experiments were extracted and analyzed for anionic and neutral PFAS to evaluate any transformation products that might be formed. No detectable levels of anionic and neutral PFAS were found in the soil of the control groups, and the soil spiked with the C4-FASA-based copolymer. The only potential metabolite found in the soil after exposure of the C8-FASA-based copolymer was methyl perfluorooctane sulfonamidoacetic acid (MeFOSAA), including both linear and branched isomers. Detection of MeFOSAA may suggest that metabolic degradation by microorganisms in the soil has taken place. The average concentration (relative standard deviation, %) of linear MeFOSAA was 0.36 ng/g dw (5%) and the branched isomers 0.09 ng/g dw (15%). The measured concentrations of the C8-FASA-based copolymers in the exposed soil after 28 days were, in some cases, higher than the theoretical value. The initial spiked level of the technical mixture was 2000 ng/g, and the measured levels were between 1670 and 6950 ng/g. These results indicate that the C8-FASA-based copolymer was heterogeneously distributed in the soil. Since the soil was measured to have a higher concentration of C8-FASA-based copolymer after the exposure test than the initial spike, no mass balance of PFAS in the soil was made, which is a limitation of this study. A better strategy in mixing these strongly sorb copolymers with the soil is needed.

Levels of two PFAAs (PFOA and PFOS) were found in the earthworms from the control groups and extraction blanks, resulting in higher MDL. In addition to the copolymers (presented in 3.1.1, Table S10) several PFAS were observed in the exposed earthworms. After 28 days of exposure to the C4-FASA-based copolymer, two metabolites: perfluorobutane sulfonamide (FBSA, 3.2–3.7 ng/g of dw) and perfluorobutanesulfonic acid (PFBS, 1.1–1.6 ng/g of dw), were detected in the earthworms. The earthworms exposed to the C8-FASA-based copolymer showed detectable levels of four metabolites, including both branched and linear isomers. The metabolites were ethyl perfluorooctane sulfonamidoacetic acid (EtFOSAA, with the total concentration of both linear and branched forms at 0.27–0.32 ng/g dw), total MeFOSAA (11–9.8 ng/g dw), total perfluorooctane sulfonamide (FOSA, 2.9–3.7 ng/g dw), and linear FOSAA (0.15–0.24 ng/g dw).

**Figure 2:**
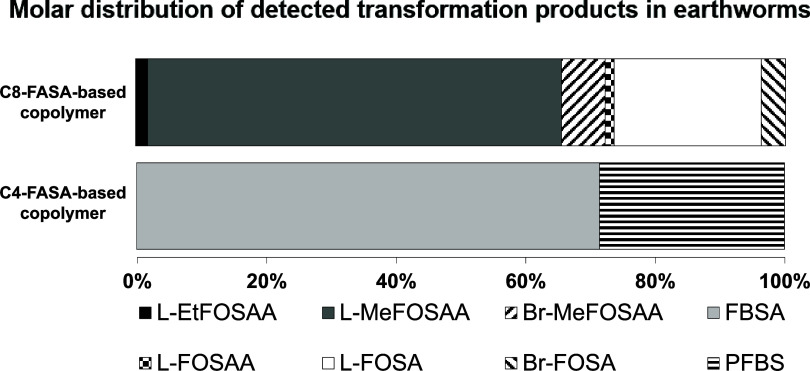
Molar distribution of detected transformation products of C8-FASA-based or C4-FASA-based copolymer in earthworms after exposure for 28 days of exposure.

Figure 2 shows the biotransformation products found in earthworms after 28 days of exposure to the FASA-based copolymer. The main transformation products for the C4-FASA-based copolymer were FBSA (71% of the detectable transformed products) followed by PFBS (29%, Figure 2). The corresponding C8-chain amide (FOSA) was observed in both linear (23%) and branched isomers (4%) in the earthworms exposed to the C8-FASA-based copolymer. Similar biotransformation products of linear FOSA and FBSA have been reported in a previous *in vitro* metabolic study on these FASA-based copolymers.^23^ Detection of both linear and branched FOSA isomers in the present study can be a result of structural isomers of the C8-FASA-based copolymer as proposed in a previous study.^24^

The C8-FASA-based copolymer has been identified (CAS no: 21055–88–9),^24^ and the proposed structure has two EtFOSA as side-chains (Figure S1), whereas the C4-FASA-based copolymer has MeFBSA side-chains. The transformation products of EtFOSA in earthworms have been observed to be FOSAA, FOSA, and PFOS^38^ and a similar biotransformation pathway could be expected for MeFBSA. Elevated levels of PFBS in earthworm after exposure to the C4-FASA-based copolymer was observed and supported this pathway. Several FOSAAs (FOSAA 1%, MeFOSAA 71%, and EtFOSAA 2%) were found in the earthworms after exposure to the C8-FASA-based copolymer, but PFOS concentrations were below the MDL (Table S10). The reason why PFOS was below MDL might be due to a slow transformation demonstrated in other studies^38−40^ or the higher MDL for PFOS in the present study.

The largest proportion of the detectable transformation products for the C8-FASA-based copolymer was MeFOSAA, at 64% for the linear isomers and 7% for the branched isomers. The reason MeFOSAA was found to be the main transformation product is still unknown. Previous biotransformation studies on ethyl perfluorooctane sulfonamido ethyl phosphate diester (DiSAmPAP), a precursor of PFOS with EtFOSA side-chains, observed perfluorooctanesulfonamidoethanol (EtFOSE), EtFOSAA, FOSAA, EtFOSA, FOSA, and PFOS as transformation products.^41,42^ The metabolic pathway for DiSAmPAP is presented in Figure S1, together with the proposed transformation pathway for the C8-FASA-based copolymer.

The reason why the present metabolic pathway does not align with the DiSAmPAP is still unclear but might be due to several hypotheses. First, the side chains of the copolymer do not have the same attachment (i.e., a urethane linkage) as DiSAmPAP; the differences in attachment might result in different transformation products. Second, the transformation product, identified as MeFOSAA, could also be of a different structure with the same precursor ion and product ion (Figure S2), giving the same *m*/*z*, and the differences in arrangements are not distinguishable on a mass spectrum. Other plausible scenarios might be demethylation or impurities present in the technical mixture. However, no other targeted PFAS showed detectable levels in the technical mixture except the copolymer itself. To investigate other plausible unknown transformation products not monitored during the target analysis, suspect screening was applied using LC-QTOF-MS in both negative and positive ionization modes, together with a mass defect analysis; however, no recognizable unknown peaks having fluorine moieties were observed. One thing to bear in mind is that sample preparation, including extraction and cleanup processes, may also lead to a loss of less stable intermediate transformation product. Further, phase I and phase II metabolisms are not mutually exclusive processes and enzymatic transformation works on multiple sites of the molecules that make identification of transformation pathway difficult.

#### 3.1.3 Accumulation of FASA-Based Copolymers in Earthworms

The accumulation potential of the copolymers was expressed by the BAF values. The BAF values were calculated for both copolymers, assuming that a steady state had been reached in the earthworms. Due to the heterogenicity in the soil, the estimation of the BAF values was done using two approaches: one taking the theoretical concentration in the soil (stated as theoretical BAF) and the other one taking the measured concentration in the soil (stated as measured BAF). All measured and theoretical BAF values are reported in Tables S11. No BAF values were found to be above one; theoretical: 0.009–0.012 and 0.38–0.87, versus the measured: 0.009–0.015 and 0.24–0.46, for the C4- and C8-FASA-based copolymers, respectively (Table S11). The average values for the C4-FASA-based copolymer were found to be 50 times lower than that of the C8-FASA-based copolymer (Table S11). The BAF values of the C8-FASA-based copolymer are in the same range as PFOA, with a carbon chain length of eight (C8).^29^ The higher BAF values for the C8-FASA-based copolymer might suggest it to be more bioavailable to earthworms compared with the C4-FASA-based copolymer. The reason for the higher accumulation may be the longer fluorinated side-chain length in the C8-FASA-based copolymer, which aligns with laboratory studies investigating the accumulation potential of PFAAs in earthworms.^28,43^ The explanation for the lower accumulation ability of the FASA-based copolymers compared to other PFAAs^28,29^ might be their large molecular weight. Another explanation can be the fact that the FASA-based copolymers were shown to degrade to other transformation products, unlike PFAAs, which dissuade their accumulative potential. Other factors influencing bioavailability for the FASA-based copolymers can be the compounds’ functional group, structure, and exposure concentrations. Decreasing accumulation potential with increasing exposure levels have been observed.^29,43^ The present study only estimated the accumulation for the FASA-based copolymers at one relevant concentration, a level that has been found in Swedish sludge.^25^ However, since the steady state was not confirmed, the causes cannot be verified. Further investigation is required to understand if the accumulation of FASA-based copolymers is concentration-dependent. To the best of our knowledge, this is the only bioavailability study on FASA-based copolymers; more knowledge is necessary to further evaluate their bioaccumulation kinetics, mechanisms, and factors affecting the rate of uptake in terrestrial organisms. More studies are needed to understand the bioaccumulation kinetics and transformation mechanisms for PFAS overall, where a major knowledge gap regarding precursors to PFSAs and PFCAs exists, e.g., other types of fluorinated polymers.

### 3.2 Field Study

#### 3.2.1 Fate of FASA-Based Copolymers on an Agricultural Field with Long-Term Application of Sludge

The results of the laboratory study demonstrate that these FASA-based copolymers are bioavailable to earthworms and can be further metabolized to other PFAS. A plausible environmental fate would be terrestrial organisms taking up these compounds when sludge is applied as fertilizer in agricultural fields and subsequently transforming the copolymers into other PFAS. Both C8- and C4-FASA-based copolymers were found in the sludge that was applied on the farmland at 0.11–0.16 and 5.5–6.6 ng FSC eq/g dw, respectively (Table S13). The measured levels is in line with our earlier study from other areas in Sweden, with total concentrations of FASA-based copolymers ranging from 1.4 to 22 ng FSC eq/g dw.^25^

No FASA-based copolymers were detected in the soil before sludge was applied (<0.02 and <0.03 ng FSC eq/g dw, respectively). However, after application of sludge, both copolymers were found in both the soil receiving no sludge and the soil with sludge application (Table S13). A notable elevation of C8-FASA-based copolymer in sludge-amended soil (0.9 ng FSC eq/g dw) compared to nontreated soil (0.25 ng FSC eq/g dw) was observed, indicating that application of sludge is an exposure pathway of the C8-FASA-based copolymer to the environment. No observable difference in levels of C4-FASA-based copolymer between soil with/without application of sludge was seen (0.040 versus 0.043 ng FSC eq/g dw).

**Figure 3:**
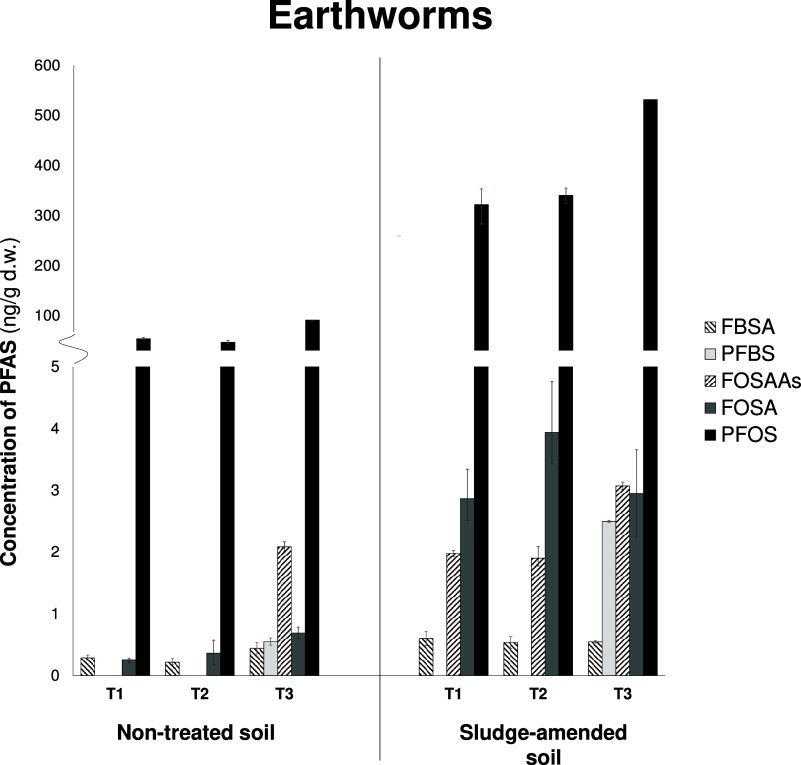
Average concentration (ng/g d.w.) and contribution of potential transformation products of FASA-based copolymers (FBSA, PFBS, FOSAAs, FOSA, and PFOS) in earthworms exposed to nontreated soil and sludge-amended soil. Error bars represent the minimum and maximum levels for the replicates. T1–T3 represent three different sampling points during the field study, i.e., T1–before application of sludge, T2–2 weeks after application of sludge, and T3–after harvesting the crops, approximately one year later. The PFAS included in FOSAAs are linear FOSAA, MeFOSAA, EtFOSAA, and Br-MeFOSAA.

In all field test groups (with/without application of sludge), no FASA-based copolymers were detected in the earthworms. The reason might be the lower concentration range of the copolymers in the field soil compared to the exposure test: for example, 0.9 FSC eq/g dw versus 16 FSC eq/g dw for the C8-FASA-based copolymer. Another explanation is that earthworms might have transformed the FASA-based copolymers to other PFAS or the FASA-based copolymers might not be stable in natural climate conditions that might have transformed into other forms. Other stability tests are necessary to give further insights. Numerous anionic and neutral PFAS (30 of 44 PFAS analyzed) were detected in both earthworms taken from nontreated soil and sludge-amended soil. The levels of all measured PFAS classes are provided in Table S12. The sum of all PFAS was in a range of 80 to 742 ng/g dw; where the PFSAs predominated the PFAS profile for all earthworms’ samples, with levels between 50 and 583 ng/g dw. The concentration for the earthworms living in the nontreated soil was five times lower compared to the earthworms living in sludge-amended soil. The PFAS levels in earthworms living in the nontreated soil were in the same range as earthworms taken from skiing resorts in Norway,^44−46^ whereas levels in the earthworms taken from the sludge-amended soil were closer to earthworms taken close to high contamination point sources, i.e., AFFF contamination and hazardous waste facility.^47,48^ The difference between the PFAS levels in earthworms living in nontreated soil compared to those living in the sludge-amended soil implies that the application of sludge is a source of PFAS contamination. However, whether the FASA-based-copolymers are a driving factor cannot be assessed. Elevated levels between sampling time points for earthworms in both groups were observed (85 to 139 ng/g dw for earthworms living in nontreated soil versus 434 to 742 ng/g dw for earthworms living in sludge-amended soil), which may indicate a time-dependent accumulation of PFAS in earthworms; or/and transformation of precursor compounds. All FASA-based copolymers’ potential transformation products were detected in the earthworms taken from nontreated soil and sludge-amended soil where all were significantly higher in levels for the earthworms living in sludge-amended soil compared to those in the nontreated soil (Figure 3). The C4-FASA-based copolymers’ potential transformation products (FBSA and PFBS) contributed to a minor part of the total PFAS (up to 0.7%), and the C8-FASA-based copolymer (FOSA and FOSAAs) accounted for 1.4% of the total PFAS. The most abundant PFAS was PFOS, which contributed to 59–74% of all PFSAs, and based on our findings in the laboratory study might be a transformation product of the C8-FASA-based copolymer. However, several precursors to PFOS exist and can influence the levels of PFOS in earthworms. This, together with the fact that several other PFAS were detected in the earthworms, including precursors to PFCAs (e.g., FTSAs, Table S12), makes it difficult to clearly evaluate the contribution of the FASA-based copolymers to the elevated PFAS levels in earthworms from sludge-amended soil compared to the earthworms from nontreated soil.

### 3.3 Environmental Implications

This study shows that the FASA-based copolymers are bioavailable for terrestrial organisms, and these observations express the importance of understanding the availability, transformation, and accumulation of FASA-based copolymers in earthworms. To the best of our knowledge, this is the first study investigating bioavailability, bioaccumulation, and biotransformation of these copolymers in earthworms. However, several processes were not investigated in the present study, including soil transformation, kinetic data during the accumulation and elimination phases, and whether the uptake processes had reached a steady state. Further studies are needed to address these aspects of these compounds.

Our results suggest that both copolymers can biologically transform into other low molecular PFAS in earthworms, even though they are large in size and high hydrophobicity. The result of the laboratory study demonstrates that the C4- and C8-FASA-based copolymers were bioavailable to the earthworms and metabolized into FOSAAs and FOSA for the C8-FASA-based copolymer and FBSA and PFBS for the C4-FASA-based copolymer, which illustrates that both copolymers could be an important and overlooked indirect source of other PFAS in the environment. This emphasizes the importance of including these kinds of compound classes in monitoring studies. However, it should be noted that this study also experienced challenges in studying these compounds. For example, we used technical mixtures for the exposure study, and we cannot rule out the presence of other monomers or impurities in the copolymer formulation, which might influence the transformation products. Also as discussed above, extraction and purification may also lead to losses of the transformation product.

Moreover, high levels of precursors to PFSAs and PFCAs have been previous reported in sludge,^49,50^ including these FASA-based copolymers.^25^ Earthworms live and feed on the soil in the surrounding habitat and are expected to be affected by sludge applied to agricultural soil. Levels of the C8-FASA-based copolymer were seen to be induced in sludge-amended soil compared to nontreated soil; and a clear increase in PFAS levels, including the transformation products of the FASA-based copolymer, was observed in the earthworms taken from the sludge-amended soil compared to those taken from nontreated soil. These observations indicate how sludge as a fertilizer is a potential route to FASA-based copolymers’ contamination and exposure. Since precursor compounds (e.g., diPAP) have been observed to be the main contributor of the PFAS levels in sludge,^25^ other side-chain fluorinated polymers are likely to be present. One common and important discussion is about the stability of side-chain fluorinated polymers and their potential to be indirect sources of other PFAS in the environment. The results of the present study together with limited numbers of other studies^19,23,51−53^ confirm that side-chain fluorinated polymers can degrade and contribute to anionic and neutral low molecular PFAS in the environment. Only two FASA-based copolymers were investigated in the current study; since fluorinated polymers are produced in high quantities, more research on bioavailability and degradation is essential for this overlooked group of PFAS.

## Supporting Information Available

Chemical and materials, instrumental analysis, method validation, results compiled from the laboratory study, results compiled from the field study (PDF)
