# Supplementary material for: Occurrence and Fate of Fluoroalkyl Sulfonamide-Based Copolymers in Earthworms–Bioavailability, Transformation, and Potential Impact of Sludge Application
Source: Environ Sci Technol. 2024 Oct 4;58(41):18304–12. doi: 10.1021/acs.est.4c01844 (PMC11483768; doi:10.1021/acs.est.4c01844)
Supplement: Supplementary file 1 — es4c01844_si_001.pdf [file es4c01844_si_001.pdf]

Supporting Information

Occurrence and fate of fluoroalkyl sulfonamide (FASA) based copolymers in earthworms – bioavailability, transformation, and potential impact of sludge application

Felicia Fredriksson, Anna Kärrman, Ulrika Eriksson, Leo WY Yeung*

Man-Technology-Environment (MTM) Research Centre, School of Science and Technology, Örebro University, Sweden, SE-701 82

* Corresponding author

E-mail address: leo.yeung@oru.se

Document Summary: 19 pages, 2 SI figures (Figures S1 – S2), 13 SI tables (Tables S1 – S13)

Table of Contents

Chemicals and materials S3

Laboratory study: soil preparation S3

Extraction of FASA-based copolymers S4

Extraction of anionic and neutral PFAS S5

Instrumental analysis S6

Table S 1. Target PFAS listed in compound classes S6

Table S 2. LC gradient method for anionic and neutral PFAS analysis and for copolymer analysis S7

Table S 3. Instrumental settings for the three different LC-MS/MS analysis conducted on all samples S8

Table S 4. SFC gradient method for ultra-short PFAAs analysis S8

Table S 5. Instrumental settings for the SFC-MS/MS analysis S9

Method validation S9

Table S 6. The extraction efficiency (recovery) and ion suppression/ ion enhancement of FASA-based copolymers in earthworm and soil analysis S9

Table S 7 The recoveries (%) of spiked samples in the laboratory study and field study S11

Table S 8 The average recovery (%) and repeatability (%) of internal standards. S12

Results compiled from the laboratory study S13

Table S 9. Information on earthworms used in the laboratory study S13

Table S 10. Individual average PFAS concentration (ng/g d.w.) in earthworms from laboratory study S13

Figure S 1. Proposed metabolic pathways for DiSAmPAP and C8-FASA-based copolymer. S15

Figure S 2. Structure of MeFOSAA and alternative structure to MeFOSAA transformation product S15

Table S 11. Bioaccumulation factor for C8- or C4-FASA-based copolymer in exposed earthworms S15

Results compiled from the field study S16

Table S 12. Individual PFAS concentration (ng/g d.w.) in earthworms from the field study S16

Table S 13 The FASA-based copolymers concentrations in sludge, soil and sludge-amended soil. S17

References S18

Chemicals and materials

Analytical reagent grade ammonium hydroxide (NH_4OH, 25%), and sodium hydroxide (NaOH, ≥99 %), HPLC- and LCMS-grade methanol (MeOH, ≥99.8 % and ≥99.9 %), and HPLC-grade acetonitrile (AcN, ≥99.9%) were from Fisher Scientific (Ottawa, Canada). HPLC-grade acetone (≥ 99.8 %) and dichloromethane (DCM, ≥99.8 %) from Honeywell Riedel-de-Haën (Seelze, Germany), *n*-hexane (≥ 99.0 %) from Supelco, Merck (Darmstadt, Germany). Three different solid-phase extraction (SPE) cartridges were used during the experimental work: disposable silica gel columns (500mg; 3 mL, J.T. Baker®) purchased from GTF/Fisher Scientific (Gliwice, Poland), graphitized carbon columns (100 mg, 1 mL, Supelclean™ ENVI-Carb ™) from Supelco; Sigma-Aldrich (St. Louis, USA), and weak ion exchange (WAX) cartridges (Oasis WAX 60 mg, 3 mL, 30 µm) from Waters Corporation (Milford, USA). LC-MS grade ammonium acetate and Supel™ QuE Z-Sep sorbent obtained from Sigma-Aldrich (St. Louis, USA) and GHP filters (0.2 µm) purchased from Waters Corporation (Milford, USA) were used. Laboratory-produced ultrapure water (18.2 MΩ) was used throughout the experimental work.

Laboratory study: soil preparation

The artificial soil was prepared by mixing all dry ingredients (70% quartz sand, 20% kaolinite clay, 10% peat, and ~ 0.7% calcium carbonate) and then moist it to a final water content of 40 – 60% of the soil's maximum water holding capacity. Equilibrium pH and organic content were measured using a 744 pH Meter Ω Metrohm and through loss of ignition (LOI), and they were found to be 5.9 (pH) and 6.04 % (LOI). The LOI of the soil was estimated at 550 °C for 6 hours. The technical mixtures of pre-2002 or post-2002 formula was added (1 mL in

methanol, MeOH) to approximately one gram of quartz sand that was placed in the middle of each test container. It was allowed to evaporate in fume hood for 24 hours before the sand was mixed thoroughly with the pre-moistened soil, before the final water content was added to the soil. The spiked soil was prepared with a final concentration of 2000 ng/g d.w. of either the technical mixture containing C4- or C8-FASA-based copolymer.

Extraction of FASA-based copolymers

An amount of 0.2, 0.25, or 1 g freeze-dried and homogenized earthworms, sludge, or soil was extracted with ultrasonication in a 15 mL polypropylene (PP) tube. The extraction procedure was performed by adding 8 mL of the extraction solution (acetone:hexane; 50:50, v/v) to the sample, vortexed, 15 minutes of ultrasonication, and then 15 minutes of centrifugation at 6000 g. The supernatant was transferred to a new PP-tube, and the extraction procedure was repeated twice. The combined extraction aliquots were gently evaporated to dryness under nitrogen gas and reconstituted with 2 mL of dichloromethane (DCM). The extract was purified using a disposable silica gel solid-phase extraction (SPE) cartridge (500 mg, 3 mL, J.T. Baker®). In brief, the silica gel SPE cartridges were conditioned by 3 mL DCM, and the sample extract was loaded on the cartridge, followed by washing the sorbent with 6 mL DCM. The target analytes were eluted with 2 mL of acetonitrile (AcN):DCM (40:60, v:v). The eluent was collected, evaporated gently to dryness under nitrogen gas, and reconstituted with 2 mL of AcN.

The reconstituted extract was vortexed and ultrasonicated for 20 minutes before further purification. Approximately, 200 mg of Supel™ QuE Z-Sep was added to the reconstituted extract and vortex mixed for 60 s. The extract with the sorbent was gently evaporated to <1 mL under nitrogen gas, then filtered with a 0.2 μm GHP filter and transferred to a vial, before

it was further evaporated to dryness under nitrogen gas and reconstituted with 0.5 mL of MeOH. The extract was ultrasonicated for 20 minutes before analysis by liquid chromatograph coupled to a tandem mass spectrometer (LC-MS/MS).

Extraction of anionic and neutral PFAS

In the extraction method targeting the anionic and neutral PFAS (potential transformation products), an amount of 0.2 g freeze-dried and homogenized earthworms was used. Mass-labeled standards were added, followed by alkaline digestion (2 mL of 0.2 M sodium hydroxide in MeOH), and thereafter extracted with MeOH, based on Powley et al. (2005) The extraction was repeated three times (2 x 4 mL and 2 mL MeOH), and the extraction aliquots were combined. The combined extract was purified by using graphitized carbon SPE columns (100 mg, 1 mL, Supelclean™ ENVI-Carb ™). The ENVI-Carb ™ SPE cartridges were conditioned by 4 mL MeOH before the sample extract was loaded on the cartridge and collected.

The extract was concentrated to <1 mL under nitrogen gas and then diluted with ultrapure water (18.2 MΩ, 10:90, v:v), to further purify the extract with Oasis weak anion exchange (WAX) SPE cartridges (60 mg, 3 mL, 30 µm, Waters Corporation, Milford, USA). In brief, the Oasis WAX SPE cartridges were preconditioned by 4 mL 0.1% ammonium hydroxide (NH_4OH) in MeOH, 4 mL MeOH, and 4 mL ultrapure water. The sample extract was loaded on the cartridge, followed by washing the sorbent with 4 mL ultrapure water, 4 mL ammonium acetate buffer (pH ≤ 4), and 4 mL MeOH:ultrapure water (20:80, v:v). The target analytes were eluted with 4 mL of 0.1% NH_4OH in MeOH. The eluent was collected, evaporated gently to 0.5 mL under nitrogen gas, and then transferred to LC-vials. The extract was ultrasonicated for 20 minutes before LC-MS/MS analysis.

Instrumental analysis

Table S 1. Target PFAS listed in compound classes (FASAs, FOSEs, FOSAAs, PFSAs, FTSs, and PFCAs), their precursor ion, chosen product ion for quantification, qualification ion, and its corresponding internal standard

Acronym	*Name*	***Precursor ion** (m/z)*	***Product ions quantification** (m/z)*	***Product ions qualification** (m/z)*	***Corresponding Internal Standard***
**Perfluorosulfonamides (FASA)**					
FBSA	Perfluorobutane sulfonamide	297.90	77.92	118.94	$^{13}C_3$ PFBS
MeFBSA	Methyl perfluorobutane sulfonamide	311.97	111.93	218.95	$^{13}C_4$ PFOS
FHxSA	Perfluorohexane sulfonamide	397.90	77.92	168.94	$^{18}O_2$ PFHxS
MeFHxSA	Methyl perfluorohexane sulfonamide	411.97	168.93	318.96	$^{13}C_4$ PFOS
FOSA	Perfluorooctane sulfonamide	497.90	78.00	168.96	$^{13}C_8$ FOSA
MeFOSA	Methyl perfluorooctane sulfonamide	512.00	169.00	-	$^{2}H_3$ MeFOSA
EtFOSA	Ethyl perfluorooctane sulfonamide	526.00	169.00	-	$^{2}H_5$ EtFOSA
**Perfluorosulfonamidoacetic acids (FOSAAs)**					
FOSAA	Perfluorooctane sulfonamidoacetic acid	555.84	497.82	418.85	$^{2}H_5$ EtFOSAA
MeFOSAA	Methyl perfluorooctane sulfonamidoacetic acid	569.77	418.86	482.76	$^{2}H_3$ MeFOSAA
EtFOSAA	Ethyl perfluorooctane sulfonamidoacetic acid	555.84	497.82	418.85	$^{2}H_5$ EtFOSAA
**Perfluoroalkyl sulfonic acids (PFSAs)**					
TFMS	Trifluoromethane sulfonic acid	179.12	79.91	98.95	$^{13}C_3$ PFBS
PFEtS	Perfluoroethane sulfonic acid	198.80	79.80	98.90	$^{13}C_3$ PFBS
PFPrS	Perfluoropropane sulfonic acid	248.90	79.90	98.90	$^{13}C_3$ PFBS
PFBS	Perfluorobutane sulfonic acid	298.90	98.90	79.96	$^{13}C_3$ PFBS
PFPeS	Perfluoropentane sulfonic acid	348.90	98.96	79.96	$^{13}C_3$ PFBS
PFHxS	Perflurohexane sulfonic acid	398.90	98.90	119.01	$^{18}O_2$ PFHxS
PFHpS	Perfluoroheptane sulfonic acid	448.97	98.90	79.96	$^{13}C_4$ PFOS
PFOS	Perfluorooctane sulfonic acid	498.90	98.96	79.96	$^{13}C_4$ PFOS
PFNS	Perfluorononane sulfonic acid	548.90	98.96	79.96	$^{13}C_4$ PFOS
PFDS	Perfluorodecane sulfonic acid	598.90	98.90	79.96	$^{13}C_4$ PFOS
PFDoDS	Perfluorododecane sulfonic acid	698.90	98.90	79.96	$^{13}C_4$ PFOS
**Fluorotelomer sulfonic acids (FTSAs)**					
4:2 FTSA	4:2 Fluorotelomer sulfonic acid	327	307	81	$^{13}C_2$ 4:2 FTSA
6:2 FTSA	6:2 Fluorotelomer sulfonic acid	427	407	81	$^{13}C_2$ 6:2 FTSA
8:2 FTSA	8:2 Fluorotelomer sulfonic acid	527	507	81	$^{13}C_2$ 8:2 FTSA
10:2 FTSA	10:2 Fluorotelomer sulfonic acid	627	607	81	$^{13}C_2$ 8:2 FTSA
12:2 FTSA	12:2 Fluorotelomer sulfonic acid	727	707	81	$^{13}C_2$ 8:2 FTSA
14:2 FTSA	14:2 Fluorotelomer sulfonic acid	827	807	81	$^{13}C_2$ 8:2 FTSA
**Perfluoroalkyl carboxylic acids (PFCAs)**					
TFA	Trifluoroacetic acid	112.90	68.96	-	$^{13}C_1$ TFA
PFPrA	Perfluoropropanoic acid	162.97	118.90	-	$^{13}C_4$ PFBA
PFBA	Perfluorobutanoic acid	212.97	169.00	-	$^{13}C_4$ PFBA
PFPeA	Perfluoropentanoic acid	262.97	219.00	-	$^{13}C_3$ PFPeA
PFHxA	Perfluorohexanoic acid	312.97	269.00	188.95	$^{13}C_2$ PFHxA
PFHpA	Perfluoroheptanoic acid	362.97	319.00	168.97	$^{13}C_4$ PFHpA
PFOA	Perfluorooctanoic acid	412.97	369.00	168.97	$^{13}C_4$ PFOA
PFNA	Perfluorononanoic acid	462.99	419.00	219.00	$^{13}C_5$ PFNA
PFDA	Perfluorodecanoic acid	512.97	469.00	219.00	$^{13}C_2$ PFDA
PFUnDA	Perfluoroundecanoic acid	562.97	519.00	268.99	$^{13}C_2$ PFUnDA
PFDoDA	Perfluorododecanoic acid	612.97	569.00	168.96	$^{13}C_2$ PFDoDA
PFTrDA	Perfluorotridecanoic acid	662.90	619.00	168.96	$^{13}C_2$ PFDoDA
PFTDA	Perfluorotetradecanoic acid	712.90	669.00	168.97	$^{13}C_2$ PFTDA
PFPeDA	Perfluoropentadecanoic acid	762.97	719.00	168.97	$^{13}C_2$ PFTDA
PFHxDA	Perfluorohexadecanoic acid	812.90	769.00	168.96	$^{13}C_2$ PFHxDA
PFHpDA	Perfluoroheptadecanoic acid	862.97	819.00	168.97	$^{13}C_2$ PFHxDA
PFOcDA	Perfluorooctadecanoic acid	912.90	869.00	168.96	$^{13}C_2$ PFHxDA

**FASA-based copolymers**					
C8-FASA-based	Bis (2-(ethyl((heptadecafluorooctyl) sulphonyl) amino)ethyl) (4-methyl-1,3-phenylene)biscarbamate	1315.10	525.90	744.00	-
C4-FASA-based	n/a	1634.20	311.90	219.00	-

n/a - not available

Liquid chromatograph coupled to a tandem mass spectrometer

A liquid chromatograph coupled to a tandem mass spectrometer (LC-MS/MS) was used for analysis of all target analytes with the exception of ultra-short-chain PFAAs. In order to achieve a good chromatographic separation for the target analytes an C18 BEH column (1.7 µm, 2.1 mm x 100 mm; Waters Corporation, Milford, USA) held in a fixed temperature at 50 °C was used with a gradient elution. A flow rate of 0.3 mL/min was set with the mobile phases consisted of 2 mM ammonium acetate in Milli-Q water: MeOH (70:30; A) and MeOH (B). For the FOSAAs analysis 5 mM 1-methylpiperidine was used as an additional additive. Detailed description of the LC methods and the source settings is given in Table S2 and S3.

Table S 2. LC gradient method for anionic and neutral PFAS analysis and a separated LC gradient method for the C8- and C4-FASA-based copolymers analysis

Anionic and neutral PFAS			***C8- and C4-FASA-based copolymers***		
**t** (min)	**Mobile phase A** (%)	**Mobile phase B** (%)	**t** (min)	**Mobile phase A** (%)	**Mobile phase B** (%)
0.00	99	1	0.00	99	1
0.57	99	1	0.57	99	1
13.00	0	100	2.00	50	50
14.00	0	100	8.00	0	100
14.20	99	1	15..00	0	100
17.00	99	1	15.20	99	1
			17.00	99	1

Table S 3. Instrumental settings for the three different LC-MS/MS analysis conducted on all samples

UPLC-TQ-S MS/MS	Target PFAS*	FOSAAs	C8- and C4-FASA-based copolymers
**Source temperature:**	150°C	150°C	150°C
**Desolvation temp:**	400°C	200°C	400°C
**Desolvation gas flow:**	800 L/h	800 L/h	800 L/h
**Cone gas flow:**	150 L/h	150 L/h	150 L/h
**Capillary voltage:**	0.70 kV	2.90 kV	3.00 kV

* = target PFAS includes the following classes FASAs, PFSAs, PFCAs and FTSAs

Supercritical fluid chromatograph coupled to a tandem mass spectrometer

A supercritical fluid chromatograph coupled to a tandem mass spectrometer (SFC-MS/MS) was used for analysis of ultra-short-chain PFAAs, including carbon chain length two and three (C2-C3) of perfluoroalkyl carboxylic acids (TFA, PFPrA) and C1-C3 perfluoroalkyl sulfonic acids (TFMS, PFPrS, PFEtS). In order to achieve a good chromatographic separation a DIOL column (1.7 µm, 3.0 mm x 150 mm; Waters Corporation, Milford, USA) held in a fixed temperature at 35 °C. A gradient elution was used with the mobile phases consisted of CO_2 (A) and 0.1 % NH_4OH MeOH (B). Detailed description of the SFC method and the source settings is seen in Table S4 and S5.

Table S 4. SFC gradient method for ultra-short PFAAs analysis

	**Ultra-short chain PFAAs**		
**t** (min)	**Mobile phase A** (%)	**Mobile phase B** (%)	**Flow rate** (mL/min)
0.00	98	2.0	1.3
8.00	40	60	0.8
9.00	40	60	0.8
9.10	98	2.0	0.8
10.20	98	2.0	1.0
11.00	98	2.0	1.3

Table S 5. Instrumental settings for the SFC-MS/MS analysis

UPLC-TQ-S MS/MS	Ultra-short chain PFAAs
**Source temperature:**	150°C
**Desolvation temp:**	350°C
**Desolvation gas flow:**	650 L/h
**Cone gas flow:**	150 L/h
**Capillary voltage:**	0.70 kV

Method validation

FASA-based copolymers in earthworm and soil

The extraction efficiency and matrix effect of the target analytes in earthworms and soil were evaluated by spiking the target analytes prior and after extraction in multiple replications. For the FASA-based copolymers in earthworms a spike recovery tests (300 ng and 50 ng) were conducted with PFAS-free laboratory grown earthworms. For the soil, the FASA-based were evaluated by a spike recovery tests (300 ng) with PFAS-free artificial soil. The extraction efficiency was estimated by the peak area of the sample spiked prior extraction divided with the peak area of the sample spiked post extraction, with subtractions of the peak area of the non-spiked sample. The matrix effects were estimated by the peak area of the sample spiked post extraction subtracted with the non-spiked sample peak area divided by the batch standard peak area. All recovery of the extraction procedures can be seen in Table S6. Validation for the extraction procedure to sludge samples have been described in Fredriksson et al. (2022).

Table S 6. The extraction efficiency (recovery) and ion suppression/ ion enhancement of FASA-based copolymers in earthworm and soil analysis

	**Earthworm**		**Soil**
**Spike Amount**	**300 ng**	**50 ng**	**300 ng**
**Compound**	(n = 3)	(n = 2)	(n = 3)
**C8-FASA-based copolymer**			
Recovery (%)	92	76	27
Ion suppression (-) or enhancement (+)	-45	-24	+45
**C4-FASA-based copolymer**			
Recovery (%)	68	56	72
Ion suppression (-) or enhancement (+)	-59	-54	-34

Anionic and neutral PFAS in earthworm

The validation of each extraction batch and sample was conducted with a spiked matrix quality control (QC) sample (Table S7) and the recoveries of the internal standards (Table S8). The reported levels included had a recovery within the range of 50 to 125 for all internal standards (mass labeled analytes spiked prior the extraction) with corresponding recovery standard (mass labeled analytes spiked post the extraction). A recovery outside this range was accepted in those cases surrogate recovery standards were used and the QC samples showed a recovery within 75 – 125%, these have been denoted with * in table S7. The recoveries of internal standards that were calculated using a surrogate recovery standard (Table S8), and native compounds quantified using a surrogate internal standard (Table S7) are denoted with ** in the tables.

Table S 7 The recoveries (%) of spiked samples in the laboratory study and field study

QC	Laboratory study		Field study
	**Earthworms** (*n* =3)	**Soil** (*n* = 3)	**Earthworms** (*n* = 3)
**Compound**	Recovery %		
FBSA**	62	108	95
MeFBSA**	108	163	95
FHxSA**	N.D.	86	23
MeFHxSA**	N.D.	37	287
L- FOSA	104*	96*	124*
MeFOSA	106*	100*	N.D.
EtFOSA	136	101*	N.D.
L-MeFOSAA	101	100	101
L-EtFOSAA -	112	107	126
TFMS**	62	105	60
PFEtS**	108	103	88
PFPrS**	27	106	39
PFBS	113*	100	104*
PFPeS**	109	99	154
PFHxS	104	103	99
PFHpS**	133	103	121
PFOS	107	105	110
PFNS**	77	96	74
PFDS**	65	103	57
PFDoDS**	81	116	35
4:2 FTSA	-	-	95
6:2 FTSA	-	-	105
8:2 FTSA	-	-	106
10:2 FTSA**	-	-	54
TFA	86	103	N.D.
PFPrA**	103	90	87
PFBA	94	96	107
PFPeA	92	100	98
PFHxA	124	104	101
PFHpA	114*	102	107*
PFOA	111	104	100
PFNA	108	100	107
PFDA	121	114	103
PFUnDA	104	101	101
PFDoDA	116*	103	102
PFTrDA**	104	159	70
PFTDA	108*	100*	100*
PFHxDA	113*	96	97*
PFOcDA**	122	231	188

N.D. – not detected

Table S 8 The average recovery (%) and repeatability (%) of internal standards added to all samples in the laboratory study (earthworms and soil) and field study (earthworms).

	Laboratory study		Field study Earthworms		
	Earthworms (n = 9)	Soil (n = 18)	T1 (n = 9)	T2 (n = 9)	T3 (n = 7)
Compound	Recovery % (Repeatability %)				
$^{13}C_8$ FOSA*	36 (10)	104 (6.8)	40 (28)	17(37)	21 (38)
$^{2}H_3$ MeFOSA*	32 (52)	443 (9.0)	N.D.	N.D.	N.D.
$^{2}H_5$ EtFOSA*	22 (31)	603 (10)	N.D.	N.D.	39 (40)
$^{2}H_3$ MeFOSAA*	125 (1.9)	96 (21)	102 (30)	91 (31)	76 (28)
$^{2}H_5$ EtFOSAA*	116 (4.0)	107 (19)	100 (30)	92 (28)	75 (30)
$^{13}C_3$ PFBS*	131 (34)	94 (4.6)	89 (7.3)	90(6.2)	132 (24)
$^{18}O_2$ PFHxS	93 (5.8)	95 (3.3)	91(12)	87(14)	88 (19)
$^{13}C_4$ PFOS	96 (1.5)	95 (4.0)	79(23)	73(25)	64 (34)
$^{13}C_2$ 4:2 FTSA*	-	-	79(7.4)	75(8.1)	65 (13)
$^{13}C_2$ 6:2 FTSA*	-	-	147(11)	140(9.9)	169(9.7)
$^{13}C_2$ 8:2 FTSA*	-	-	118(24)	108(22)	114(32)
$^{13}C_4$ PFBA	95 (3.8)	94 (3.0)	88(3.0)	81(5.7)	85 (9.0)
$^{13}C_3$ PFPeA	92 (1.8)	95 (2.7)	89 (4.4)	87(4.7)	88 (8.3)
$^{13}C_2$ PFHxA	98 (3.5)	96 (2.9)	91 (2.5)	88 (5.0)	88 (7.9)
$^{13}C_4$ PFHpA*	34 (51)	94 (6.5)	7 (16)	8 (12)	4 (24)
$^{13}C_4$ PFOA	95 (2.0)	95 (2.7)	93 (2.9)	94 (2.7)	96 (5.1)
$^{13}C_5$ PFNA	96 (2.9)	95 (3.2)	93 (4.5)	91 (6.9)	86 (6.2)
$^{13}C_2$ PFDA	95 (5.3)	96 (3.7)	88 (11)	83 (14)	83 (11)
$^{13}C_2$ PFUnDA	92 (3.7)	95 (3.5)	78 (19)	73 (19)	71 (22)
$^{13}C_2$ PFTDA*	179 (17)	131 (15)	42 (47)	24 (65)	25 (44)
$^{13}C_2$ PFDoDA*	176 (17)	84 (18)	30 (44)	34 (23)	28 (31)
$^{13}C_2$ PFHxDA*	212 (25)	387 (43)	144(50)	68 (91)	71 (52)

N.D. – not detected

Results compiled from the laboratory study

Table S 9. Information on earthworms (before and after exposure) included in control, Pre-2002 and Post-2002 tests

		Control			Pre-2002			Post-2002		
**Before exposure**	*n* (earthworms)	10	10	10	10	10	10	10	10	10
	Average weight (g)	0.49	0.47	0.49	0.49	0.49	0.50	0.49	0.49	0.49
	SD of weight (g)	0.10	0.09	0.08	0.08	0.09	0.11	0.10	0.09	0.10
**After exposure**	*n* (earthworms)	10	10	10	10	10	10	10	10	9
	Average weight (g)	0.66	0.65	0.61	0.60	0.61	0.64	0.60	0.59	0.64
	SD of weight (g)	0.13	0.07	0.15	0.09	0.08	0.22	0.11	0.09	0.15
	Water content (%)	79.9	79.1	78.6	75.5	79.3	79.4	78.6	77.9	77.4
	n (juveniles)	10	11	20	8	16	6	9	18	2

Table S 10. Individual average PFAS concentration (ng/g d.w.) and relative standard deviation in earthworms after exposure of C8-FASA-based (Pre-2002) and C4-FASA-based (Post-2002) copolymers. Concentrations above method detection limit (MDL) are stated together with the analytes MDL. Higher MDL is stated for PFOA and PFOS due to concentrations found in the control groups.

			Laboratory study	
**Class**	**Analyte**	**MDL**	**Pre-2002**	**Post-2002**
**Perfluorosulfonamides** (FASA)	**FBSA**	0.33	<0.33	3.5 (8)
	**MeFBSA**	0.25		
	**FHxSA**	0.25	N.Q.	N.Q.
	**MeFHxSA**	0.25		
	**L- FOSA**	0.1	2.9 (13)	
	**Br-FOSA**	0.25	0.48 (18)	
	**MeFOSA**	0.25	N.Q.	N.Q.
	**EtFOSA**	0.25	N.Q.	N.Q.
**Perfluorooctane sulfonamidoacetic acids** (FOSAAs)	**FOSAA**	0.1	0.2 (20)	
	**L-MeFOSAA**	0.48	9.4 (5)	<0.48
	**Br-MeFOSAA**	0.25	0.99 (15)	
	**L-EtFOSAA**	0.1	0.28 (10)	
	**Br-EtFOSAA**	0.25		
**Perfluoroalkyl sulfonic acids** (PFSAs)	**TFMS**	0.25		
	**PFEtS**	0.25		
	**PFPrS**	0.25		
	**PFBS**	0.1		1.4 (18)
	**PFPeS**	0.1		
	**PFHxS**	0.1		
	**PFHpS**	0.1	N.Q.	N.Q.
	**PFOS**	1.1	<1.1	<1.1
	**PFNS**	0.1		
	**PFDS**	0.1		
	**PFDoDS**	0.25	N.Q.	N.Q.
**Fluorotelomer sulfonic acids** (FTSAs)	**4:2 FTSA**	0.25		
	**6:2 FTSA**	0.25		
	**8:2 FTSA**	0.25		
	**10:2 FTSA**	0.25		
	**12:2 FTSA**	0.25		

	**14:2 FTSA**	0.25		
**Perfluoroalkyl carboxylic acids** (PFCAs)	**TFA**	0.25		
	**PFPrA**	0.25		
	**PFBA**	0.25		
	**PFPeA**	0.1		
	**PFHxA**	0.1		
	**PFHpA**	0.1		
	**PFOA**	0.55	<0.55	<0.55
	**PFNA**	0.1		
	**PFDA**	0.1		
	**PFUnDA**	0.25		
	**PFDoDA**	0.25		
	**PFTrDA**	0.25		
	**PFTDA**	0.25		
	**PFPeDA**	0.25		
	**PFHxDA**	0.25		
	**PFHpDA**	0.25		
	**PFOcDA**	0.25	N.Q.	N.Q.
**Side-chain fluorinated copolymers** (ngFSCeq./g)	**C8-FASA-based**	0.10	11.4 (39)	<0.10
	**C4-FASA-based**	0.16	<0.16	0.78 (29)

N.Q. – not quantified

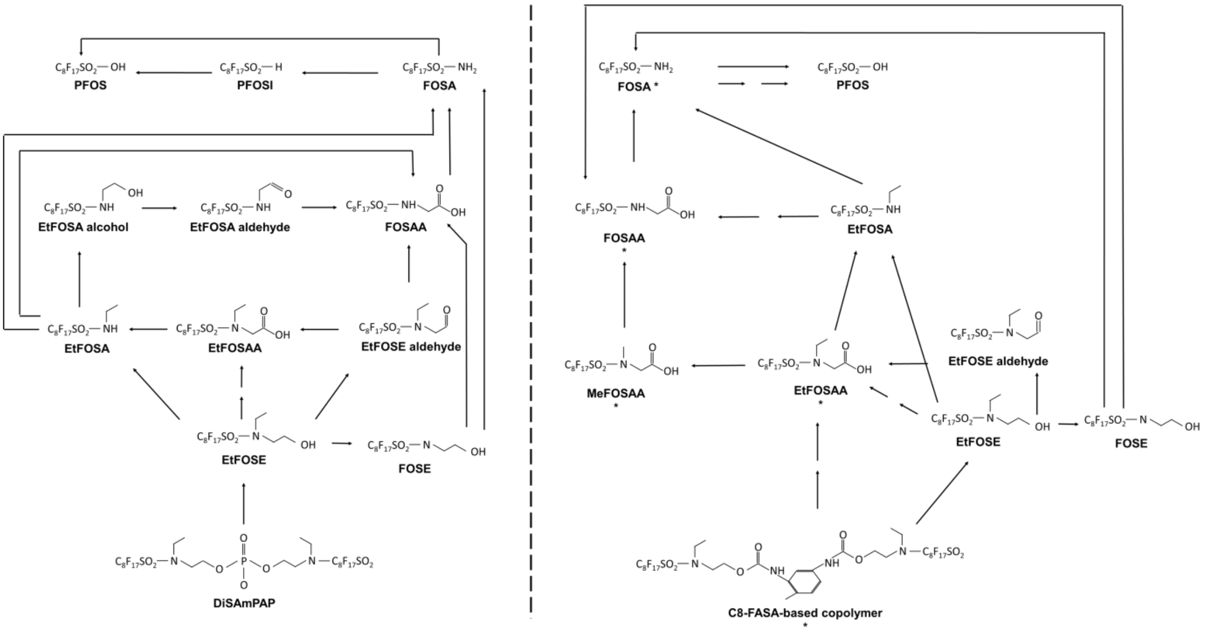

Figure S 1. Proposed metabolic pathways for the biotransformation of DiSAmPAP (left) and C8-FASA-based copolymer (right). The DiSAmPAP transformation is suggested in previous studies,(Avendaño and Liu 2015, Gaillard et al. 2017, Zhang et al. 2017, Zhang et al. 2018) and C8-FASA-based copolymer is based on the present exposure study on earthworms; the analytes denoted with * were detected in the earthworms and is identified as a transformation product.

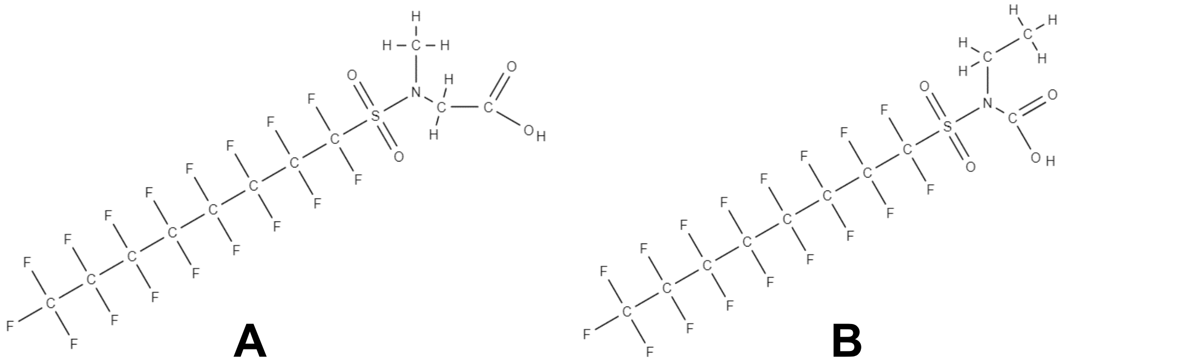

Figure S 2. A) Structure of transformation product MeFOSAA, B) alternative structure to MeFOSAA transformation product (EtFOSA based structure containing a carboxylic acid moiety)

Table S 11. Bioaccumulation factor (BAF) for C8- or C4-FASA-based copolymer in exposed earthworms; including both the theoretical BAFs – taking the theoretical/spiked concentration in the soil into account, and measured BAFs -taking the measured concentration in the soil in account. Each value is based on analysis of each test vessel consisting of ten earthworms.

	BAF ($kg_{dw,soil} * kg_{dw,worm}^{-1}$)					
	**Theoretical**			**Measured**		
**C8-FASA-based**	0.84	0.38	0.87	0.24	0.45	0.31
**C4-FASA-based**	0.012	0.009	0.017	0.009	0.012	0.016

Results compiled from the field study

Table S 12. Individual PFAS concentration (ng/g d.w.) in earthworms from the field study for three time-points (T1-T3), living in non-treated or sludge-amended soil. Only concentrations above method detection limit (MDL) are stated together with the analytes MDL. The concentrations are displayed as average (relative standard deviation).

		**Non-Treated Soil**			**Sludge-Amended Soil**		
**Analyte**	**MDL**	**T1**	**T2**	**T3**	**T1**	**T2**	**T3**
**Perfluorosulfonamides** (FASA)							
**FBSA**	0.25	0.28 (15)	0.22 (25)	0.44	0.60 (17)	0.54 (18)	0.55
**MeFBSA**	0.25	N.Q.	N.Q.	N.Q.	N.Q.	N.Q.	N.Q.
**FHxSA**	0.25	N.Q.	N.Q.	N.Q.	N.Q.	N.Q.	N.Q.
**MeFHxSA**	0.25	N.Q.	N.Q.	N.Q.	N.Q.	N.Q.	N.Q.
**L- FOSA**	0.1	0.25 (17)	0.36 (50)	0.69	2.9 (15)	3.9 (18)	2.9
**MeFOSA**	0.25	N.Q.	N.Q.	N.Q.	N.Q.	N.Q.	N.Q.
**EtFOSA**	0.25	N.Q.	N.Q.	N.Q.	N.Q.	N.Q.	N.Q.
**Perfluorooctane sulfonamidoacetic acids** (FOSAAs)							
**FOSAA**	0.25			1.3	0.54 (1.5)	0.69 (17)	
**L-MeFOSAA**	0.25				0.64 (3.3)	0.78 (13)	1.4
**L-EtFOSAA**	0.25				1.4 (5.1)	1.1 (6.4)	1.3
**Perfluoroalkyl sulfonic acids** (PFSAs)							
**TFMS**	0.25						
**PFEtS**	0.25						
**PFPrS**	0.25	N.Q.	N.Q.	N.Q.	N.Q.	N.Q.	N.Q.
**PFBS**		N.Q.	N.Q.	0.5	N.Q.	N.Q.	2.5
**PFPeS**							6.1
**PFHxS**		3.4 (7.1)	2.4 (11)	2.9	6.3 (17)	8.1 (4.7)	28
**PFHpS**		0.72 (22)	0.98 (20)		3.6 (10)	5.5 (11)	13
**PFOS**		54 (3.8)	47 (7.0)	91	322 (11)	341 (4.5)	532
**PFNS**							
**PFDS**					1.7 (15)	0.97 (18)	1.7
**PFDoDS**	0.25						
**Fluorotelomer sulfonic acids** (FTSAs)							
**4:2 FTSA**	0.25			0.22			0.19
**6:2 FTSA**	0.1	1.2 (36)	0.69 (5.7)	<	3.9 (4.8)	5.1 (5.6)	<
**8:2 FTSA**	0.1	0.2 (11)	0.17 (11)	0.38	1.8 (2.5)	2.7 (2.5)	5.6
**10:2 FTSA**	0.25	0.28 (26)	0.26 (27)	0.47	2.6 (4.2)	2.1 (28)	4.2
**12:2 FTSA**	0.25				0.94 (14)	0.19 (35)	0.49
**14:2 FTSA**	0.25						
**Perfluoroalkyl carboxylic acids** (PFCAs)							
**TFA**	0.25	N.Q.	N.Q.	N.A.	N.Q.	N.Q.	N.A.
**PFPrA**	0.25			N.A.			N.A.
**PFBA**	0.25		3.5 (4.0)	2.5		4.9 (4.0)	4.9
**PFPeA**	0.1		1.1 (7.9)		2.5 (4.6)	8.8 (2.9)	9.1
**PFHxA**	0.1		3.0 (5.3)	1.5	3.3 (5.1)	9.3 (1.2)	9.8
**PFHpA**	0.1	1.5 (30)	3.0 (13)	2.4	2.7 (23)	11 (3.5)	13
**PFOA**	0.1	2.6 (3.1)	4.5 (2.3)	3.5	7.9 (2.0)	25 (1.2)	26
**PFNA**	0.1	1.4 (3.2)	1.0 (6.4)	1.4	2.5 (8.1)	5.3 (6.5)	6.0
**PFDA**	0.1	1.5 (12)	1.3 (2.9)	2.3	6.1 (1.8)	7.8 (6.5)	12

**PFUnDA**	0.1	1.3 (6.2)	0.92 (13)	2.2	3.4 (0.93)	3.1 (1.7)	5.2
**PFDoDA**	0.25	3.1 (7.8)	1.8 (9.4)	4.3	13 (4.0)	11 (4.7)	14
**PFTrDA**	0.25	7.1 (10)	2.2 (24)	7.2	12 (9.3)	5.3 (15)	5.3
**PFTDA**	0.25	4.8 (7.0)	3.5 (5.6)	7.8	23 (1.5)	22 (5.3)	25
**PFPeDA**	0.25		1.2 (16)	3.5	4.2 (6.0)	3.1 (20)	5.9
**PFHxDA**	0.25	1.0 (8.7)	0.90 (17)	1.9	4.8 (3.3)	5.3 (20)	5.25
**PFHpDA**	0.25		0.26 (15)	3.5	0.83 (8.0)	1.1 (11)	0.79
**PFOcDA**	0.25	N.Q.	N.Q.	N.Q.	N.Q.	N.Q.	N.Q.
**Side-chain fluorinated copolymers** (ngFSCeq./g)							
**C8-FASA-based**	0.10	<0.10	<0.10	<0.10	<0.10	<0.10	<0.10
**C4-FASA-based**	0.16	<0.16	<0.16	<0.16	<0.16	<0.16	<0.16

N.Q. – not quantified
N.A. – not analyzed

Table S 13 The C8- and C4-FASA-based copolymers concentrations (ng FSC eq./g d.w.) in sludge (n = 3), soil (n = 2) and sludge-amended soil (n = 2). The soil samples are collected at two time points (T1 and T3). The relative standard deviation is expressed in between the brackets.

	Sludge	Non-Treated Soil		Sludge-Amended Soil	
**Analyte**		**T1**	**T3**	**T1**	**T3**
**Side-chain fluorinated copolymers** (ngFSCeq./g)					
**C8-FASA-based**	0.13 (23)	<0.02	0.09	<0.02	0.26
**C4-FASA-based**	6.5 (9.7)	<0.03	0.04	<0.03	0.04

References

Avendaño, S. M. and J. Liu. Production of PFOS from aerobic soil biotransformation of two perfluoroalkyl sulfonamide derivatives. *Chemosphere.* **2015**, *119*, 1084-1090 DOI: 10.1016/j.chemosphere.2014.09.059.

Fredriksson, F., A. Kärrman, U. Eriksson and L. W. Yeung. Analysis and characterization of novel fluorinated compounds used in surface treatments products. *Chemosphere.* **2022**, *302*, 134720 DOI: 10.1016/j.chemosphere.2022.134720

Gaillard, J., B. Veyrand, M. Thomas, X. Dauchy, V. Boiteux, P. Marchand, B. Le Bizec, D. Banas and C. Feidt. Tissue uptake, distribution, and elimination of perfluoroalkyl substances in juvenile perch through perfluorooctane sulfonamidoethanol based phosphate diester dietary exposure. *Environ. Sci. Technol.* **2017**, *51*(13): 7658-7666 DOI: 10.1021/acs.est.6b05598.

Powley, C. R., S. W. George, T. W. Ryan and R. C. Buck. Matrix effect-free analytical methods for determination of perfluorinated carboxylic acids in environmental matrixes. *Anal. Chem.* **2005,** *77*(19): 6353-6358 DOI: 10.1021/ac0508090.

Zhang, L., L. S. Lee, J. Niu and J. Liu. Kinetic analysis of aerobic biotransformation pathways of a perfluorooctane sulfonate (PFOS) precursor in distinctly different soils. *Environ. Pollut.* **2017**, *229*: 159-167 DOI: 10.1016/j.envpol.2017.05.074.

Zhang, S., H. Peng, D. Mu, H. Zhao and J. Hu. Simultaneous determination of (N-ethyl perfluorooctanesulfonamido ethanol)-based phosphate diester and triester and their biotransformation to perfluorooctanesulfonate in freshwater sediments. *Environ. Pollut.* **2018**, *234*: 821-829 DOI: 10.1016/j.envpol.2017.12.021.
